# Supplementary material for: Exploring the Effect of Different Storage Conditions on the Aroma Profile of Bread by Using Arrow-SPME GC-MS and Chemometrics
Source: Molecules. 2023 Apr 20;28(8):3587. doi: 10.3390/molecules28083587 (PMC10141652; doi:10.3390/molecules28083587)
Supplement: Supplementary file 1 [file molecules-28-03587-s001.zip › molecules-2283564-supplementary.pdf]

Exploring the Effect of Different Storage Conditions on the Aroma Profile of Bread by Using Arrow-SPME GC-MS and Chemometrics

Supplementary Materials

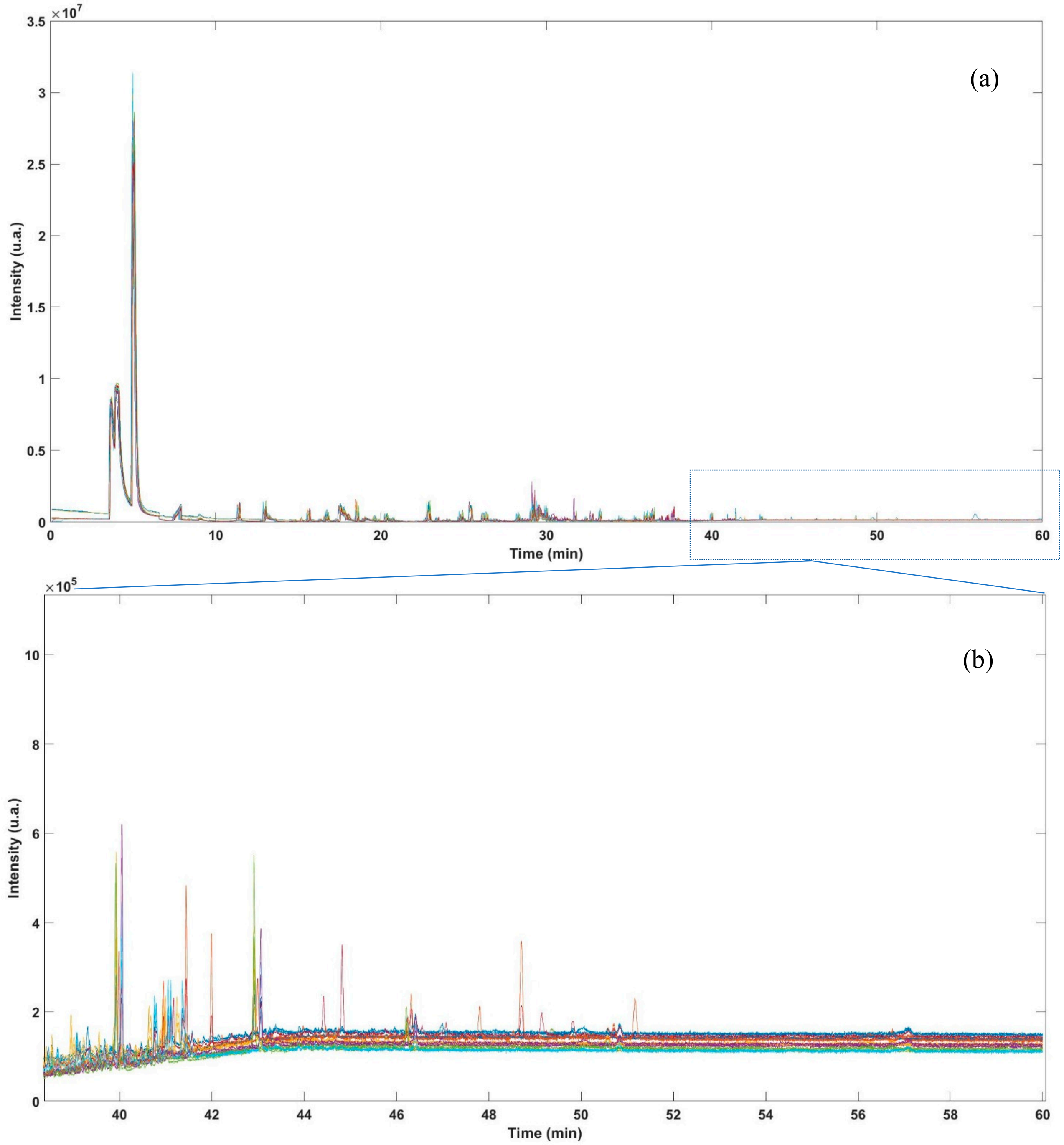

Figure S1. All the obtained chromatograms (a) and a zoom of the last 23 minutes (b) where no analytes belonging to bread were detected.

Table S1. Areas of the peaks of the identified analytes.

**Sample**	Diacetyl	2-butanone, 3-hydroxy-	2-heptanone	Ethanone, 1-(2-furanyl)-	6-methyl-5-hepten-2-one	Acetic acid	Hexanoic acid
A_0	362580	555893	197915	352039	58132	3062839	169002
A_1FZ	374589	548673	254111	377352	63995	3040931	213487
A_3FZ	341944	548980	263262	375494	69112	3118551	215801
A_4FZ	260743	465326	236315	294346	67134	2735400	167805
A_1fr	350876	621831	49549	262216	58488	3558877	247135
A_3fr	393928	630692	60536	210904	158293	2980029	293639
A_4fr	330002	697219	45232	254242	79712	3273853	267250
B_0	773379	1848177	70164	416871	63930	3766845	276530
B_1FZ	743306	2044581	82449	371301	53387	3870499	211202
B_3FZ	835441	2195406	83271	405386	65107	3862852	269444
B_4FZ	629244	1864938	87292	400012	69872	3505012	237479
B_1fr	868041	2327111	32369	260598	58199	3889462	225703
B_3fr	761559	2084191	43229	228855	91255	3795751	211424
B_4fr	774880	2024986	32087	167631	57409	3513581	209587
C_0	791992	2227236	150040	151549	69939	3362607	204116
C_1FZ	1002979	2738813	180761	188356	74964	4000679	286176
C_3FZ	1104546	2616418	197667	197531	82091	3804543	300115
C_4FZ	830456	2495837	194949	226792	96344	3711470	403555
C_1fr	1003238	3035506	50046	110271	53816	3988048	318178
C_3fr	948380	2585271	50252	114142	94090	3794203	324330
C_4fr	899969	3018179	62366	167703	69654	3768533	403414

**Sample**	Acetic acid, ethyl ester	Propanoic acid, 2-hydroxy-, ethyl ester	3-methyl-butanoic acid, ethyl ester	3-hexen-1-ol, acetate	Acetic acid, hexyl ester	Butyl glycol acetate	Octanoic acid, ethyl ester
A_0	155457	73585	69530	228146	97397	10774	83802
A_1FZ	333326	75257	86014	256806	112391	8560	95968
A_3FZ	555297	77678	94805	264530	117495	6845	94055
A_4FZ	608043	60164	85558	221611	95659	6079	77590
A_1fr	16822	87278	5478	60100	29542	8573	68270
A_3fr	31886	78439	8446	35429	18125	14770	63646
A_4fr	52929	99674	6179	47691	22894	14315	81068
B_0	105942	244905	6919	154215	66215	13442	247892

B_1FZ	106893	279351	5685	129339	55236	11447	164469
B_3FZ	108928	327126	7572	134478	58287	8222	156177
B_4FZ	154227	248761	8048	140981	57313	11126	163469
B_1fr	16111	310178	1793	42480	20684	10113	144483
B_3fr	47703	259068	2273	33804	17184	6393	116848
B_4fr	53281	259508	2907	21562	11500	6320	101563
C_0	71475	286994	8440	101045	38483	46332	143621
C_1FZ	76863	374890	7185	114584	45810	51359	161698
C_3FZ	101260	343027	7838	122825	48254	58241	167900
C_4FZ	103509	313034	6992	139536	55741	73939	220674
C_1fr	23775	381364	1251	27217	12113	32530	107995
C_3fr	43934	325195	1354	24226	10945	34143	111993
C_4fr	54942	360431	2589	47268	21906	37890	125007

**Sample**	Ethyl hexanoate	1-hexanol	2-furanmethanol	2-methyl-1-butanol	3-methyl-1-butanol	2-methyl-1-propanol	Benzeneethanol
A_0	95418	507145	683240	327158	1238479	516815	1366524
A_1FZ	113224	450793	603445	345063	1349725	513738	1446524
A_3FZ	124877	597197	616721	432419	1934583	585216	1596076
A_4FZ	108219	416632	419656	380374	1764166	524878	976473
A_1fr	32054	433838	715264	316707	1224556	500020	1514120
A_3fr	28986	319982	607363	214049	947474	325995	1429647
A_4fr	44506	520122	719828	299093	1417492	433905	1595819
B_0	77719	322407	268233	252193	941663	370372	2816750
B_1FZ	75658	274592	402428	272780	981862	361146	1721689
B_3FZ	87451	322335	554858	279330	1169738	314293	2357311
B_4FZ	93709	226668	255241	262212	1090696	329744	1885703
B_1fr	33623	286744	444092	234287	783271	290523	1846807
B_3fr	32151	249877	448331	207273	856435	285613	1529826
B_4fr	24686	238610	419320	183969	766727	245241	1414325
C_0	172966	441671	177464	276956	967387	329054	1835376
C_1FZ	209445	553938	235422	339695	1235334	349651	2375968
C_3FZ	237972	533775	211123	346876	1412797	371591	2469960

C_4FZ	279506	491540	174069	347828	145543<br>9	377424	3311696
C_1fr	54220	412448	258048	260291	888434	300162	2042570
C_3fr	45984	383347	218558	226601	897031	275038	2184315
C_4fr	70868	452196	238320	236872	100658<br>1	275108	2663185

**Sample**	3-methyl-butanal	Heptanal	Hexanal	2-heptenal	Benzaldehyde	Octanal	2-Octenal
A_0	24282	357960	647319	49250	1790989	94685	37849
A_1FZ	48064	388674	891847	97654	2011452	120846	49711
A_3FZ	64257	401980	837726	86362	2099720	134679	43928
A_4FZ	61420	343009	804414	96455	1800671	141222	39118
A_1fr	10805	199036	318403	88830	734112	173263	46391
A_3fr	10090	219242	510333	88421	592143	370410	57169
A_4fr	22376	184237	365352	148180	755487	184365	58318
B_0	105931	167430	460459	120699	1924786	91198	71159
B_1FZ	134017	176191	491363	92491	1575193	97638	47387
B_3FZ	137766	178869	661862	72531	1593681	98824	49876
B_4FZ	141614	164784	462490	48137	1786272	96748	37755
B_1fr	20654	122646	200001	31841	626494	154518	36397
B_3fr	28571	155064	290949	44630	655337	240068	37278
B_4fr	26661	105999	278077	60680	380656	172576	36222
C_0	38537	223724	605861	51099	1074899	104950	39972
C_1FZ	61891	270728	800411	84522	1252668	136945	46106
C_3FZ	62800	284435	713934	32390	1281025	146637	36599
C_4FZ	53658	263690	654699	62412	1662900	168420	50965
C_1fr	12596	127712	819511	69539	412227	187075	63497
C_3fr	14443	186385	449143	40667	486538	287366	45268
C_4fr	28034	187532	931308	89023	723342	269715	61231

**Sample**	Nonanal	2-nonenal	Decanal	Limonene	2-methyl-2-undecanethiol	2(3H)-furanone, dihydro-5-pentyl-	Methyl-pyrazine
A_0	479024	47897	88160	106614	335734	110772	184687
A_1FZ	586199	42121	66534	124184	409472	148683	171290
A_3FZ	619024	44902	60202	132057	424623	115480	166383
A_4FZ	999154	28860	165274	112465	393570	97899	139216
A_1fr	1010245	53877	187602	68972	214460	139181	155565
A_3fr	1806750	94449	424795	60072	200619	131933	128995
A_4fr	1049396	50973	186806	135393	164720	131203	159565

B_0	831991	87368	45896	59660	718123	118164	204413
B_1FZ	725963	49821	68010	53865	589007	83529	227460
B_3FZ	733933	76885	54888	59816	572851	99590	235325
B_4FZ	674681	55402	96750	57565	600085	92531	241953
B_1fr	977132	83667	176948	55208	301359	85626	222726
B_3fr	1405651	82434	248655	108681	239751	81387	202463
B_4fr	1182061	57558	193751	85109	225583	92895	152761
C_0	502618	39281	53242	29763	546012	115798	66177
C_1FZ	687936	37429	70037	34081	564760	138095	84824
C_3FZ	899488	51086	82873	39570	605904	111711	99462
C_4FZ	877351	45229	130793	49360	707902	217767	111059
C_1fr	920103	47784	159449	35048	375752	118628	74462
C_3fr	1942504	76753	274306	74285	279426	150472	81619
C_4fr	1536303	56221	219939	147121	288170	158254	114290

**Sample**	Furfural	2,5-dimethyl-pyrazine	Ethyl hexanoate	2-pentyl-furan
A_0	683471	121182	95418	396233
A_1FZ	670077	131595	113224	355126
A_3FZ	676373	128411	124877	399722
A_4FZ	545933	100559	108219	169175
A_1fr	536378	103822	32054	111920
A_3fr	437855	92722	28986	136056
A_4fr	526613	137631	44506	76305
B_0	1272700	371382	77719	545777
B_1FZ	1200152	331099	75658	465843
B_3FZ	1176635	375959	87451	714213
B_4FZ	1125905	365399	93709	696107
B_1fr	847843	250942	33623	226299
B_3fr	804756	224110	32151	186717
B_4fr	557267	168262	24686	166527
C_0	442679	163259	172966	664524
C_1FZ	508985	190647	209445	561191
C_3FZ	526258	207770	237972	1101940
C_4FZ	535936	252888	279506	956056
C_1fr	317645	123453	54220	187137
C_3fr	338458	128580	45984	206468
C_4fr	411866	172955	70868	190951

Table S2. Weighed masses of each sample.

**Sample**	**Mass (g)**
A_0	1.0326 ± 0.0001

A_1FZ	1.0280 ± 0.0001
A_3FZ	1.0272 ± 0.0001
A_4FZ	1.0204 ± 0.0001
A_1fr	1.0421 ± 0.0001
A_3fr	1.0226 ± 0.0001
A_4fr	1.0384 ± 0.0001
B_0	1.0391 ± 0.0001
B_1FZ	1.0512 ± 0.0001
B_3FZ	1.0225 ± 0.0001
B_4FZ	1.0362 ± 0.0001
B_1fr	1.0283 ± 0.0001
B_3fr	1.0273 ± 0.0001
B_4fr	1.0244 ± 0.0001
C_0	1.0446 ± 0.0001
C_1FZ	1.0237 ± 0.0001
C_3FZ	1.0751 ± 0.0001
C_4FZ	1.0253 ± 0.0001
C_1fr	1.0248 ± 0.0001
C_3fr	1.0237 ± 0.0001
C_4fr	1.0358 ± 0.0001
